# Characterizing the genetic basis of bacterial phenotypes using genome-wide association studies: a new direction for bacteriology

**DOI:** 10.1186/s13073-014-0109-z

**Published:** 2014-11-22

**Authors:** Timothy D Read, Ruth C Massey

**Affiliations:** Department of Medicine, Division of Infectious Diseases, Emory University School of Medicine, Atlanta, GA 30322 USA; Department of Human Genetics, Emory University School of Medicine, Atlanta, GA 30322 USA; Department of Biology and Biochemistry, University of Bath, Bath, BA2 7AY UK

## Abstract

Genome-wide association studies (GWASs) have become an increasingly important approach for eukaryotic geneticists, facilitating the identification of hundreds of genetic polymorphisms that are responsible for inherited diseases. Despite the relative simplicity of bacterial genomes, the application of GWASs to identify polymorphisms responsible for important bacterial phenotypes has only recently been made possible through advances in genome sequencing technologies. Bacterial GWASs are now about to come of age thanks to the availability of massive datasets, and because of the potential to bridge genomics and traditional genetic approaches that is provided by improving validation strategies. A small number of pioneering GWASs in bacteria have been published in the past 2 years, examining from 75 to more than 3,000 strains. The experimental designs have been diverse, taking advantage of different processes in bacteria for generating variation. Analysis of data from bacterial GWASs can, to some extent, be performed using software developed for eukaryotic systems, but there are important differences in genome evolution that must be considered. The greatest experimental advantage of bacterial GWASs is the potential to perform downstream validation of causality and dissection of mechanism. We review the recent advances and remaining challenges in this field and propose strategies to improve the validation of bacterial GWASs.

## Introduction

Genome-wide association studies (GWASs) involve testing large numbers of genetic variants, usually single nucleotide polymorphisms (SNPs) or insertions and deletions (indels), within a population of individual organisms for statistically significant associations with a given phenotype [1]. The first successful GWAS in humans, published in 2005, examined a set of 96 patients with age-related macular degeneration, a condition that leads to loss of vision in older adults, and 50 matched controls [2]. Out of 116,204 SNPs tested, two were statistically significantly associated with the condition. One of the SNPs was found in the complement factor H gene, encoding a protein integral to host immunity, and the condition has since then been linked to autoimmunity [3]. Although there is some controversy about specific aspects of the approach [4], many GWASs have now been published, making hundreds of associations between SNPs and important human diseases [5].

**Figure 1:**
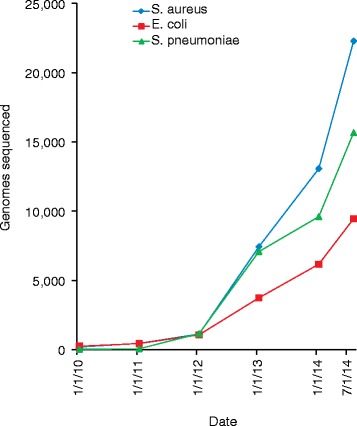
**Growth in the cumulative number of next-generation sequencing runs in public databases for three important bacterial pathogens,** ***Staphylococcus aureus*** **,** ***Escherichia coli*** **and** ***Streptococcus pneumoniae*** **.** The data were acquired by querying the National Center for Biotechnology Information Short Read Archive database and excluding datasets linked to RNA-seq experiments.

GWASs are clearly an important tool for genetic analysis but their use in microbiological research has been relatively slow to emerge [6]. Smaller-scale genetic association studies in bacteria have been performed for a number of years. Early research used PCR and limited sequence data (for example, data from multi-locus sequence typing [7]) or comparative genome hybridization [8] to link bacterial phenotypes with the presence or absence of specific genes or with the clonal background of an isolate [9–14]. In human genetics, high-throughput genotyping of panels of common SNPs using microarrays and bead-based assays have been a mainstay for GWASs for the past 10 years [15]. The creation of SNP-typing panels is, however, generally associated with high fixed costs and so few platforms were custom-designed for bacterial species. Those that were designed for bacteria were practically limited to species with low nucleotide diversity (such as *Bacillus anthracis* [16]). This reality began to change in 2010 with the advent of large-scale genome sequencing using affordable and accurate data produced by Illumina HiSeq and MiSeq instruments. These instruments made generation of the whole genome sequence of 50 or more bacterial strains a routine experiment and opened the door for bacterial GWASs (Figure 1).

The first successful application of a GWAS to bacteria using shotgun sequence data was published in 2013 [17] (see Table 1). Sheppard *et al.* [17] used a novel association approach to probe the genetic factors responsible for host adaptation in 192 shotgun-sequenced *Campylobacter jejuni* and *C. coli* strains. In another publication in the same year, mutations in *Mycobacterium tuberculosis* genes responsible for resistance to anti-tuberculosis drugs were detected on the basis of their recurrent appearance in resistant lineages of a whole-genome phylogenetic tree [18]. Three studies published in 2014 have extended the use of GWASs on bacterial shotgun data. Laabei *et al*. [19] studied a collection of 90 methicillin-resistant *Staphylococcus aureus* clinical isolates and identified more than 100 polymorphisms that associated with the ability of the bacteria to lyse human cells. Alam *et al*. [20], also studying *S. aureus*, used a GWAS to determine mutations in the RNA polymerase *rpoB* gene that are significantly associated with the clinically important vancomycin-intermediate-resistant phenotype. The first GWAS to use a number of cases and controls on the scale commonly seen in human genetic research was recently published by Chewapreecha *et al*. [21]; these researchers sequenced 3,701 *Streptococcus pneumoniae* isolates to identify polymorphisms associated with beta-lactam resistance.

**Table 1:** **Early bacterial genome-wide association studies based on whole-genome shotgun data**

**Organism**	**Sample size**	**Phenotype**	**Finding**	**Genome-wide association study program used**	**Reference**
	**(isolates)**				
*Campylobacter jejuni* and *C. coli*	192	Host adaptation	Vitamin B5 biosynthesis is important	30 bp ‘word’ searching [17]	[17]
*Mycobacterium tuberculosis*	123	Antibiotic resistance	39 novel resistance-associated loci	PhyC [18]	[18]
*Staphylococcus aureus*	75	Antibiotic resistance	Novel associated single nucleotide polymorphism in *rpoB* gene	ROADTRIPS [50]	[20]
*S. aureus*	90	Virulence	121 novel associated loci	PLINK [49]	[19]
*Streptococcus pneumoniae*	3,701	Antibiotic resistance	Multiple novel associated loci	PLINK [49]	[21]

What is made clear by even these few early studies is that a GWAS is a powerful first step towards characterizing a phenotype at a population level. It is an unbiased screening approach to discover new loci that correlate with a specific phenotype. GWASs can form the basis of studies of the functionality of regulatory pathways and expression mechanisms and, when performed robustly, can be used to build predictive tools for the translation of genomic data into the clinical microbiology setting. Bridging the gap between genomics and traditional molecular genetics has the potential to uncover untapped levels of detail on how bacteria survive and cause disease. Discoveries could be used to personalize medicine so that treatments can be tailored for individual patients on the basis of the genome sequence of the infecting microbe. In this review, we discuss what should be taken into account when planning a bacterial GWAS, how to improve the validation of GWASs, how these studies are likely to impact on clinical microbiology in the future and what challenges remain.

## Design considerations for bacterial GWASs

Bacterial GWAS is a brand new field. It is increasingly easy to generate genomic data, but there are challenges in identifying optimum GWASs strategies. Some of these challenges are also shared with eukaryotic GWASs, and, although there are many experiences and tools that can be drawn from eukaryotic studies (Table 2), caution should be used when translating approaches developed for different domains of life.

**Table 2:** **Similarities and differences between bacterial and eukaryotic genome-wide association study approaches**

**Feature**	**Bacteria**	**Eukaryote**
Ploidy	Haploid	Diploid
Genetic re-assortment	Infrequent short gene conversion and horizontal gene transfer events	Homologous recombination and chromosome segregation linked to reproduction
Accessory (non-core) genes	Variable numbers in different species	Rare
Linkage disequilibrium	Variable across the genome and between species	Variable across the genome
Population structure	Asexual, generally highly structured, except for relatively rare homologous recombination events	Sexual, variable allele frequencies in subpopulations owing to non-random mating, ancestral divergence, drift
Confounders in genome-wide association studies	Population structure	Population structure
How to move from association to causality	Genetic reconstruction of mutations in laboratory strains, transposon mutant screens	Forward genetics in animal models or cultured tissue systems; linkage to known genetic diseases; large monogenic association studies
Current burden of proof for causality	Molecular Koch’s Postulates	Combined genetic and experimental evidence

There are several prerequisites for a successful bacterial GWAS. There must be a testable phenotype and a set of bacterial strains with whole-genome sequences. Experimenters need to choose a statistical analysis strategy and perform power calculations to ensure that there are enough strains in their study to have a reasonable chance of successful association. None of these prerequisites are truly independent of one another.

### Phenotypes

It is necessary to consider whether the phenotype to be tested by the GWAS is a continuously varying quantitative phenotype or a binary case versus control trait. A continuous phenotype can be subdivided into discrete categories, for instance using accepted breakpoints for antibiotic sensitivity to resistance [20]. Phenotypes for bacterial GWASs (such as host species, infection type, severity, or outcome) can be gleaned from metadata collected at the time of isolation of the strain or obtained by experimentation. It is important to make assessments about the consistency of the annotation, especially when the data come from multiple sources. In the case of experimental phenotypes, the need to perform the assays on very large numbers of strains will tend to limit experiments to those phenotypes that can be assayed in a simple and relatively inexpensive way. For these reasons, the early studies have concentrated on phenotypes such as antibiotic resistance [18,20,21] and *in vitro* toxicity [19].

In considering the genetic basis of the phenotype, it is important to have an idea of the effect sizes: a measure of the correlation of the variant with the phenotype. Effect sizes vary from 0 to 1, with 1 meaning that the phenotype is completely explained by the variant. Many bacterial variants (such as antibiotic-resistance mutations) are assumed to have very large effects, akin to a Mendelian trait in eukaryotes, because they are necessary for the survival of the cell. However, bacterial phenotypes that are influenced mainly by low-effect variants surely exist, and the use of GWASs is probably the only feasible approach to determining their genetic basis.

### Genetic variation and population structure in bacterial strains

**Figure 2:**
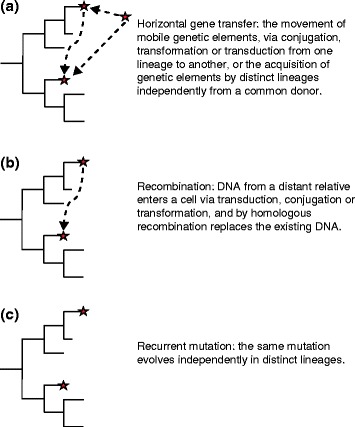
**Natural mechanisms for introducing homoplasious mutations into the genomes of bacterial populations.** Homoplasious mutations are necessary for association studies. The figure represents three mechanisms for forming an identical genetic variant (red star) on different lineages of a hypothetical phylogenetic tree of bacterial strains: **(a)** horizontal gene transfer, **(b)** recombination and **(c)** recurrent mutation.

GWASs are dependent for their success on the way the genetic variants to be tested (for example, SNPs) are distributed among the genomes of the subject population. There are distinct differences in the dynamics of genetic variation between humans (and other higher diploids) and bacteria. In humans, genetic recombination and chromosome segregation, necessary for shuffling alleles, occurs each generation. A newly occurring mutation will be genetically linked to neighboring alleles as part of the same haplotype until a recombination event occurs to break the linkage. The extent that any two alleles within a population are on the same ancestral ‘haplotype block’ of DNA is termed their linkage disequilibrium (LD) and usually decreases with genetic distance on the chromosome. This mixing of alleles between different genetic backgrounds is important for distinguishing causal loci from passively linked mutations. Asexual bacterial reproduction does not offer the opportunity to exchange genetic information this frequently. There are instead three natural mechanisms that generate the variability needed for GWASs: gene acquisition through horizontal gene transfer (HGT) and non-homologous recombination, gene conversion through homologous recombination, and recurrent mutation (Figure 2). In each case, these processes can create homoplasy, which is the presence of a similar genetic locus (SNPs, indels, genes and so on) on different branches of the phylogeny.

Insertion of complete genes as a result of HGT can generate diversity for association testing in bacteria (Figure 2a) [22]. The three classical mechanisms of HGT are transduction by bacteriophages, transformation of DNA segments, and plasmid-mediated conjugation. Genome sequencing of multiple isolates within bacterial species has given rise to the concept of a ‘pan-genome’ [23], which consists of a core of genes present in every strain and all of the accessory genes (defined as those found in some but not all members of the sequenced population). Depending on the bacterial species, accessory genes may encode virulence factors, antibiotic resistance determinants, or other loci that contribute to the adaptation of the bacterium to its environment [24]. Ideally for GWASs, these genes should be acquired multiple times by different lineages. Deletion of accessory genes is a process that is effectively the reverse of HGT in creating the variable presence of accessory genes across strains and lineages of a species [25].

In bacteria, homologous recombination happens after unidirectional transfer of DNA sequence into the recipient via HGT, leading effectively to gene conversion (Figure 2b) [26]. These events are rare, and generally do not occur at every generation, even in highly promiscuous bacterial species [27]. Exchanged DNA segments tend to be small (hundreds to a few thousand bp, although rarely larger events of more than 10 kb have been reported [28]), and typically create a patchwork of islands of introduced variation across the genome. Recombination results in a decay of LD across bacterial genomes that varies in rate in different species [29]. Several studies have shown recombination to be a mechanism used for adaptation. An example of this involves the mosaic *penA* allele XXXIV, derived from recombination between *Neisseria gonorrhoeae* and a commensal strain that confers resistance to cephalosporin antibiotics [30]. The *penA* XXXIV allele has been introduced by recombination into multiple *N. gonorrhoeae* lineages [31]. In another study that examined natural patterns of gene conversion, unidirectional transfer of DNA segments into diverse lineages was also found to be responsible for rapid adaptation to aquatic sub-niches by *Vibrio cyclitrophicus* [32].

Recurrent mutation of genetic variants within different lineages of a species as a response to selection offers a third way to create homoplasious genetic loci (Figure 2c). This can happen often in bacteria because of large local population sizes (sometimes billions of cells within a single infection). One example of a recurrent mutation is that which causes the H481Y codon change in the *rpoB* gene; this mutation has occurred in multiple *S. aureus* lineages and confers intermediate levels of resistance to vancomycin [20].

Bacterial species differ considerably in genetic diversity and show characteristic historical rates of recombination, HGT and recurrent mutation [26,27,29]. Many bacterial species are highly clonal, and exchange DNA through homologous recombination infrequently. In these species, recurrent mutation will be very important for genetic association [18]. *M. tuberculosis*, the causative agent of tuberculosis, is a classic example of a near-clonal species, with only 1.1% homoplasic SNPs within its core genome [33]. Rates of recombination (as measured by fixed events) also vary between species [27,34]. In one example, the Gram-negative pathogen *Chlamydia trachomatis*, gene conversion frequencies have been found to be higher in hotspots such as the *OmpA* major outer member protein gene [35], which is under diversifying selection for immune evasion. In *S. aureus*, horizontally transferred genes and regions surrounding them recombine at higher frequency than the core genome [36,37].

Another important aspect to consider when designing a bacterial GWAS is population structure. Populations of a species are considered to be structured if they contain a non-random distribution of alleles within subpopulations. Population structure in humans can occur through mechanisms such as genetic drift, ancestral divergence [38] and non-random mating within subpopulations [39]. The stratification of human populations is reflected in complex patterns of LD in different parts of the chromosome and in different subgroups [40]. Importantly, population structure may confound GWASs, especially if it is not recognized, by causing the appearance of higher than expected allele frequencies within certain members of the study set [41]. Problems relating to structured genetic variation would be expected to be worse in bacterial strains than in human populations as bacteria are haploid and asexual. In the absence of recombination, all fixed genetic variants will be passed on to descendants and be in LD with other mutations that occur in that lineage. The separation of causative variants from passive linked loci is potentially a difficult problem.

The problem of population structure has been addressed in bacterial GWASs by using phylogenetic approaches [18,21], by using clustering followed by permutation [19], and by using databases of known variation to identify common mutations [20]. For future experimental design, it should also be possible not only to study variation in naturally occurring populations but also to utilize laboratory-induced mutation and recombination techniques to generate banks of strains that have artificial homoplasies [42].

### Markers for bacterial GWASs

Whole genes, SNPs, indels or other loci such as mobile genetic elements [10] can be used as markers in GWASs. The quality of the DNA sequence data is an important consideration for experimental design. Because of the small genome size of bacteria it is now rare for Illumina shotgun projects to have average coverage (the number of sequence reads per base) of less than 20. At this level of redundancy, the confidence of the consensus base-calling accuracy is high [43,44]. Furthermore, the portion of the genome represented by multiple sequencing reads is also high, making the problem of imputation of missing genotypes small relative to human studies [45]. The increasing use of single molecule long-read sequencing technologies, which can produce complete or near-complete genome sequences following *de novo* assembly [46], will help to reduce the frequency of missing larger loci (such as genes or intergenic regions) in bacterial genomes.

SNPs are the most common units used as markers in GWASs. SNPs are commonly detected by comparison to a reference sequence, which can lead to ascertainment bias: the strains that are more genetically similar to the reference tend to have more accurate SNP calls. An alternative approach is to use ‘reference-free’ multiple alignment methods [47,48]. The penalty for these approaches, which use short sequence words (k-mers) for matching, is that multiple SNPs that occur in close proximity (less than the chosen word length) might not get reported. For convenience, early studies have focused on SNPs found in core regions of the genome (or in accessory genes that are found in all strains in the comparison set). Developing a strategy for the treatment of SNPs in accessory genes that are present in some strains but not in others will be important for bacterial GWASs. These are not missing data, as encountered in human projects with low sequence coverage [45]. One possible approach could be to run an association test for each accessory gene SNP using just the strains in which it occurs separate from the core genome GWAS.

An alternative to focusing on SNPs is to use k-mers. The *Campylobacter* GWAS by Sheppard *et al*. [17] used 30 bp ‘words’ extracted from the assembled genome sequences as the unit for association, each of which was tested against the species origin of isolation. The advantage of this approach was that it allowed discovery of multiple types of variants (SNP, indels and gene insertions) without requiring a genome alignment.

### Bacterial GWAS statistical analysis approaches and software

There are many tools developed for human GWASs available for porting to bacterial datasets. Some consideration of the differences between bacterial and eukaryotic genetics will be needed when assigning parameters (Table 2). The popular PLINK [49] software for regression-based association of both quantitative and case versus control studies has been used (Table 1). In the study by Chewapreecha *et al*. [21], the Cochran-Mantel-Haenzel test was used to correct for genetic background in discovering SNPs that are associated with beta-lactam resistance in two genetically different *S. pneumoniae* population clusters. Alam *et al*. [20] used ROADTRIPS [50], a regression-based approach that incorporates corrections for both known and inferred population structure.

Two phylogeny-based approaches for association have been developed specifically for bacteria. In the Predict Phenotypes From SNPs package outlined by Hall [51], SNPs were associated with phenotypic changes inferred in internal branches of the whole-genome phylogeny. This method utilized template-free genome assembly and tree construction based on the kSNP software [47]. The phylogenetic convergence or ‘PhyC’ approach [18] looked at recurrent mutations on the tips and internal nodes of the phylogenetic tree, assuming that mutations occurred recently under strong selection. Significance was tested using a permutation approach to ask whether the number of times a SNP occurred on branch leading to an antibiotic-resistant strain versus an antibiotic-sensitive strain was unusual in the population.

### Calculation of statistical power

Software that estimates statistical power allows researchers to calculate the number of cases and controls needed to have a realistic chance of rejecting the null hypothesis (that there is no association between the variant and the phenotype) when the alternative hypothesis is indeed true. For example, a calculation may yield the number of strains necessary to have an 80% chance of detecting an association with an effect size of 0.5 or greater with a *P*-value threshold of 0.05. Power calculations have been important in human GWASs for improving the experimental design to increase the probability of obtaining a statistically meaningful result [52], and there are now a myriad of software packages available to researchers [40,53,54]. Commonly included variables that tend to increase power include larger effect-size cutoff, reduced population structure, and increased sequence quality [55].

The number of genetic loci to be tested is an important variable in statistical power calculations. Multiple tests of significance increase the chances of false-positive calls. For example, if 20 randomly selected loci are tested independently at the standard 0.05 significance threshold, one locus would be expected by chance to be a false positive. A conservative Bonferroni correction for the number of hypothesis tests in the study is usually imposed in order to reduce false-positive calls. Experimental designs that reduce the number of genetic variants tested serve to increase power. One way to reduce the number of tests is to select a subpopulation of the original set strains with a smaller number of total SNPs. Other strategies include disregarding low-frequency mutations and/or mutations that cause synonymous mutations or SNPs in intergenic regions, or treating all individual mutations within a genetic feature (a gene, intergenic region and so on) as having the same aggregate effect. The risk in removing rare mutations from the study is that they may be important for the phenotype, as has been found in several human diseases. This was also the case in the Laabei *et al.* study [19] where four novel toxicity-affecting intergenic loci were identified and their effect verified by mutagenesis. Permutation tests using scrambled cases and controls can also be used to increase statistical power [21,52]. Finally, false discovery rate could be used as an alternative to significance thresholds for identifying candidate loci [56].

Simple power models [52] may have value in offering a starting point when considering study size. The experience in human genetics is that the sophistication of power statistics has increased as knowledge of the population structure has improved [40]. Because of the immense variation in bacterial species genetics, empirical calculations using simulated genome datasets may be particularly important for experimental design. A software package for designing experiments based on recurrent mutations between matched pairs of cases and controls has recently been developed [57]. From the evidence of the early bacterial GWASs (Table 1), quite a small number of cases and controls (n =75) might be required to find variants associated with phenotype that have a large effect size. Future GWASs with experimental design informed by basic studies on bacterial species population structure and involving increasingly large collections of phenotypically characterized strains may be able to unearth larger numbers of small-effect variants.

## Validating the results of GWASs: bridging the gap between genomics and traditional microbial molecular genetics

**Figure 3:**
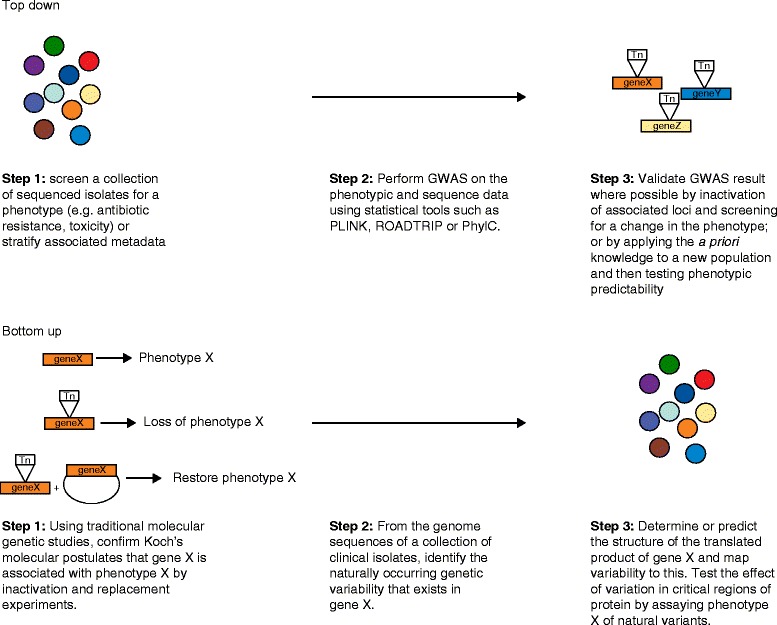
**Schematic representation of genomics/genome-wide association studies and traditional microbial genetics strategies.** The top-down approach [6] (genomics/genome-wide association studies (GWASs) typically begins with a pool of sequenced genomes and attempts to discover genes that are associated with a particular phenotype. Bottom-up approaches (molecular genetics) perform experiments to prove or disprove hypotheses about the function of particular genes or regions of the genome. These approaches can be integrated when knowledge gained from molecular genetics is used to validate unbiased GWASs- and genomics-based experiments.

GWASs on bacteria has already yielded interesting new loci that are associated with clinically important phenotypes, but how can we be confident that these associations are causative or functionally linked? This question has been examined in depth in human studies (Table 2). Significance tests implemented in GWAS software necessarily rely on assumptions, such as a lack of cryptic population structure and consistent rates of mutation across evolutionary history, that may produce higher error rates than the *P*-values suggest [41]. Experimental errors in base-calling and phenotyping could also contribute to spurious results. We know from the experience of human GWASs that some loci found to be associated with a trait can turn out to have little or no functional significance [58]. Therefore, unless the associated locus has been previously shown to affect the phenotype, functional validation is desirable [19]. The questions that surround the strategy for functional validation are part of an ongoing dialog between two apparently diametrically opposed experimental philosophies in modern microbiology: the ‘top down’ unbiased, genomics-based approaches (which include GWASs and other experimental strategies [59–62]), and the ‘bottom-up’, gene-by-gene approach of classical molecular genetics (Figure 3) [6]. The disconnect is that, on the one hand, we will eventually have thousands of genome sequences of every bacterial pathogen, whereas on the other hand, the current *modus operandi* of molecular genetics is focused on fine-scale analysis of individual proteins in a very small number of isolates. The coming of GWASs will hopefully speed the genesis of a powerful synthesis between these two approaches.

Traditional molecular genetic approaches have been instrumental in carefully dissecting the functions of thousands of bacterial genes, sometimes down to the level of highly complex interactions between host cells and pathogens that lead to disease (such as Type III secretion or superantigens [63,64]). Typically, researchers seek to design systems to examine discrete phenotypes, where upon mutation (directed or random), the loss or gain of a specific phenotype can be efficiently screened or selected. Depending on the activity of the gene in question, further specific molecular or cellular experiments follow to characterize the mechanisms in detail. This approach is tremendously powerful in manipulating the microorganism and the environment to test precise hypotheses within the artificial confines of the laboratory. Since the 1980s, the dominant paradigm for linking genes to phenotype in microbiology has been based on the Molecular Koch’s Postulates, outlined by Falkow [65]. These state that disruption and reconstruction of the gene under investigation coupled with loss and regain of the phenotype is needed for firm proof of a functional role. Molecular Koch’s Postulates are often used as a stringent standard for validation, although the original article offered a nuanced discussion of some of the difficulties in their application to all situations [65].

Validation by genetic disruption and reconstruction can be applied to GWASs results, especially for microorganisms for which genome-wide transposon mutant libraries are available, such as *S. aureus*, *Escherichia coli*, *Streptococcus pneumoniae*, *Pseudomonas aeruginosa*, *Yersinia pseudotuberculosis* and *Salmonella enterica* [60,66,67]. Nevertheless, there can be situations in which laboratory genetics are more challenging or even impossible, for example when the identified polymorphism is in an essential gene, or when the species being studied is not amenable to genetic manipulation. We are also increasingly sampling beyond where the traditional microbiology laboratory can venture, sequencing single cells [68], and reconstructing genomes directly from environmental DNA [69,70]. In these circumstances, it may be possible to use a model genetic organism such as *E. coli* to test for the phenotypic effect of a mutation, but any result may not be considered a direct validation under the Molecular Koch’s Postulates rules.

There is also the problem of potential epistatic interactions between genes and the contribution of non-core, accessory genes to the phenotype. If a reconstructed mutant strain does not have the expected phenotype, this could result from the lack of a specific interacting allele in the host strain, or possibly a missing non-core gene. No single strain can ever represent a species, but the strains commonly used for genetic reconstruction may be especially poor choices because of their long history of laboratory adaptation [71]. Laboratory strains are chosen because they are locally available and have familiar, useful properties: generally fast growth and easy genetic manipulation. As a consequence, laboratory strain phenotypes often do not represent the majority of the species. The quixotic properties of certain laboratory strains have misled generations of scientists about the true nature of their subject organisms. For example, the ubiquitous genetic workhorse, *Bacillus subtilis* 168 is a very rare naturally transformable strain within its species (it is also a non-swarming tryptophan auxotroph, amongst other unusual features [72]), and the *S. aureus* genetic strain 8325-4 has a mutation in the *sigB* locus that causes an enhanced toxic profile [73].

If the one-at-a-time genetic reconstruction method is unlikely to work for all variants discovered through GWASs, and in some cases may produce misleading results because of complex gene interactions, statistical modeling may also be able to provide an alternative type of validation. Commonly, machine-learning techniques such as support vector machines and random forests [74] can be trained on a reserved portion of the dataset and then tested on the remainder. Random forests were used to make reliable predictions of an individual isolates’ level of toxicity and vancomycin-intermediate phenotype [19,20]. Although a successful model would not be able to explain the mechanistic contribution of the loci, it would inform that sufficient information on the genetic basis of the phenotype for sensitive prediction had been learned.

Ultimately, it is likely that combining molecular genetic and statistical modeling approaches will be fruitful. In a hypothetical situation in which GWASs results in more than 200 loci that are significantly associated with a complex phenotype, validating the effect of the top 20 most important mutations might allow the statistical model to predict the phenotype accurately in more than 95% of unknown strains. There has been interest in developing methods to prioritize variants discovered in human GWASs [75], and potentially some of these approaches can be applied to the bacterial realm. Further on in the future, systems biology and systems genetics approaches to high dimensional data integration may offer an alternative to ‘one gene at a time’ genetic validation [76,77].

## How will GWASs affect clinical microbial diagnostics?

Bacterial GWASs have the potential to deepen our understanding of phenotypic variation across pathogenic species. This information will be particularly useful in the future as we attempt to interpret genome sequences that are routinely produced by clinical microbiology laboratories. There is great interest in the development of whole-genome sequencing for clinical diagnostics of pathogens [78-81] because it is possible to envisage genomics technology maturing to the extent that *de novo* sequencing becomes a relatively cheap and rapid assay. Whole-genome sequence data have numerous advantages over the directed PCR-based tests that currently dominate this arena. Unlike shotgun genomics, PCR relies on the presence of highly conserved DNA sequences for primer binding and yields false-negative results when these are mutated, as happened, for example, with a plasmid-borne marker for *C. trachomatis* [82]. Importantly, the whole-genome sequence also allows unbiased discovery of other information about the strains that the clinician may not have considered, such as the unexpected presence of antibiotic-resistance genes.

To take advantage of our ability to acquire the genome sequence of a pathogen rapidly ahead of the results of a laboratory-based phenotypic test, such as an antibiotic minimal inhibitory concentration (MIC) test, we must be able to not only call drug sensitivity on the basis of the genome sequence alone but also know the reliability of the assignment. Several schemes for predicting drug resistance have already been developed, based on knowledge obtained from early comparative genomics and genetic knockout studies [83,84]. Further development of these diagnostic tests will necessitate understanding how the activities of well known genes are influenced by epistatic interactions within the pathogen species. For the reasons we have outlined earlier, GWASs provide the natural training set data to build statistical models that predict phenotypes by integrating genetic variation across the entire genome. Another advantage of a test that is based on trained genomic data is that variability in how the phenotype is measured is no longer a problem. Many clinically relevant phenotypes are ascertained using a plethora of different technologies and are variable across different conditions. MIC, for example, can be determined by disk diffusion, test strips, spiral plating, or several other methods. GWASs performed on a genetically diverse set of strains measured using gold-standard phenotypic assays could be used to train models that effectively replace much routine clinical antimicrobial-resistance testing.

Large-scale clinical sequencing could provide a pool of thousands of new genomes for GWASs that could discover variants that have ever-smaller effect. Existing statistical models could also be tested and refined with the new clinical data. For this feedback cycle to occur, we will need to improve and make more efficient our collection of metadata (time and place of isolation, clinical manifestations, phenotype tests and so on). Several schema for organizing bacterial strain metadata have been proposed [85,86]. Even today, when it is possible to sequence 96 or more strains each day on a bench-top instrument, it is a feat of organization to manually gather metadata retrospectively for submission with the genomes to public databases. For us to keep up with future throughput, we need systems that facilitate information storage at the time of isolation and phenotypic testing. This will be a challenge, particularly in the high-throughput, time-pressured environment of the clinical microbiology laboratory. There is also an issue with access to collections of sequenced isolates. Many organizations make sequence data available in public databases, but either do not maintain the bacterial collections from which the sequenced DNA was extracted or are unable to bear the costs of making large sets of strains available to the research community. The solution is to have regular accession of large numbers of sequenced isolates with high-quality metadata from clinical and academic laboratories into public strain collections, but this will need new organization and funding.

## Conclusions and perspectives

GWAS in bacteria is a new research opportunity that is being driven forward by advances in genome-sequencing technology. Although in its infancy, the early studies have shown it to be not only a reliable method to identify loci that affect a phenotype but also a powerful tool to uncover new levels of complexity in the expression of clinically important bacterial traits. The approaches and tools used to do this are likely to adapt and develop as we sample ever-greater numbers of bacterial genomes that are associated with high-quality metadata. What is clear is that GWASs represent a versatile and highly productive approach to maximizing the usefulness of the genomic data available to us from both laboratory and clinical settings.

## Abbreviations

GWASs: Genome-wide association studies
HGT: Horizontal gene transfer
indel: Insertion and deletion
LD: Linkage disequilibrium
MIC: Minimal inhibitory concentration
PCR: Polymerase chain reaction
SNP: Single nucleotide polymorphism
